# Advances in the application of insulin resistance-related indicators in the clinical evaluation of metabolic dysfunction-associated steatotic liver disease

**DOI:** 10.3389/fgstr.2026.1859647

**Published:** 2026-09-04

**Authors:** Yi Li, Huan Li, Bolun Zhang, Qingming Wu, Hui Long

**Affiliations:** 1 Institute of Digestive Diseases, Center of Clinical Medicine, Tianyou Hospital Affiliated to Wuhan University of Science and Technology, Wuhan, China; 2 Hubei Provincial Key Laboratory of Occupational Hazard Identification and Control, Wuhan, China

**Keywords:** fatty liver, indicators, insulin resistance, MASLD, non-invasive markers

## Abstract

Metabolic Dysfunction-Associated Steatotic Liver Disease (MASLD) has become the most common chronic liver disease worldwide, and its core pathophysiological mechanism is closely related to insulin resistance. This literature review aims to systematically summarize the application progress of insulin resistance-related indicators in the clinical assessment of MASLD, points out the limitations of existing indicators in clinical application, and looks forward to future research directions, including the development of more accurate and non-invasive biomarkers, exploration of multi-omics integration models, and promotion of individualized diagnosis and treatment strategies, with the expectation of optimizing the clinical management of MASLD and improving patient prognosis.

## 1 Introduction

Metabolic Dysfunction-Associated Steatotic Liver Disease (MASLD) formerly known as Non-alcoholic Fatty Liver Disease (NAFLD), has a broad disease spectrum. It progresses from simple hepatic steatosis to Metabolic Dysfunction-Associated Steatohepatitis (MASH), and in more severe cases, it can lead to liver fibrosis, cirrhosis, and even hepatocellular carcinoma, posing a serious threat to patients’ lives and health (1, 2). MASLD has become the most common chronic liver disease worldwide, with an age-standardized prevalence of approximately 38% in adults (1, 3). The disease burden shows a distinct geographical gradient, highest in Latin America (44.4%), followed by the Middle East and North Africa (around 39%), moderate levels in Asia with significant internal variation, and lowest in Western Europe (25.1%) (1, 3). Prevalence is even higher among individuals with type 2 diabetes: reaching up to 80.6% in Eastern Europe, 71.2% in the Middle East, and 60.0% in South Asia (4). MASLD is also a growing concern among children and adolescents, with a global prevalence of 7%–14%, higher in Asian regions (15%) than in non-Asian countries (11%), and rising as high as 47% among obese children. In China, a nationwide study involving 500,000 adults revealed significant regional, occupational, age, and gender differences in MASLD prevalence. Geographically, northern China had a higher rate (41.73%) than southern China (38.93%), possibly due to dietary patterns rich in fat and carbohydrates in the north. Occupation-wise, farmers had the highest prevalence (52.1%), followed by administrative staff (46.2%), while workers had the lowest (42.8%), suggesting that physical activity may offer protective effects. Gender and age disparities were also evident: men had about twice the risk of women (53.0% vs. 18.6%), and the highest prevalence was observed in those aged 41–64 (47.0%). However, the fastest increase occurred among young people aged 18–40 between 2015 and 2023, with MASLD prevalence rising from 19.1% to 30.2%. Overall, China’s MASLD prevalence increased from 30.0% in 2015 to 43.2% in 2023, consistent with global trends (5). These data collectively indicate that MASLD prevalence is not uniformly distributed but follows a high-to-moderate-to-low pattern across Latin America/Middle East and North Africa, Asia, and Western Europe, with substantial internal variations linked to regional diets, occupations, and demographic factors. This systematic stratification is critical for developing targeted public health strategies at the regional level.

In 2023, the diagnostic criteria for MASLD were updated, and the original term “non-alcoholic fatty liver disease (NAFLD)” was renamed to “ Metabolic Dysfunction-Associated Steatotic Liver Disease (MASLD)”. This renaming more accurately reflects the metabolic pathophysiological nature of the disease and emphasizes its close connection with various metabolic disorders (1). The harm of MASLD is not confined to the liver alone. It is interrelated with various metabolic diseases such as cardiovascular disease (CVD), type 2 diabetes mellitus (T2DM), and chronic kidney disease (CKD), collectively forming a complex “cardiovascular-renal-hepatic-metabolic syndrome” (CRHM) system, indicating that it can affect multiple systems throughout the body (6). In the complex pathophysiological mechanism of MASLD, insulin resistance (IR) is widely recognized as one of the core driving factors (7–9). This article reviews the research progress, clinical application status and inherent limitations of IR-related markers in the diagnosis, severity assessment, prognosis prediction and treatment response monitoring of MASLD, explores the practical application value of IR in MASLD, and provides evidence-based support for clinical practice.

## 2 Overview of IR

Insulin resistance (IR) refers to a state in which the target cells of the body exhibit reduced responsiveness or impaired sensitivity to the normal biological effects of insulin, resulting in the need for a higher concentration of insulin to achieve its physiological functions normally (10). This condition is typically characterized by hyperinsulinemia, meaning that the pancreatic β cells need to secrete excessive insulin to maintain normal blood glucose levels in order to compensate for the decline in insulin sensitivity.

## 3 The core mechanism of IR in the onset of MASLD

IR and MASLD have a bidirectional influence on each other. MASLD is not only a manifestation of IR in the liver, but conversely, hepatic steatosis can also exacerbate systemic IR, creating a vicious cycle (9, 11, 12).

Under physiological conditions, insulin, on the one hand, reduces glucose output by inhibiting hepatic gluconeogenesis and glycogenolysis, on the other hand, it regulates hepatic lipid metabolism by promoting *de novo* lipogenesis (DNL) and inhibiting fatty acid oxidation (11, 12). However, once insulin resistance occurs, the liver becomes insensitive to the inhibitory effect of insulin on gluconeogenesis, leading to an increase in glucose release from the liver and subsequently causing hyperglycemia (9, 11). However, the liver’s function of promoting *de novo* lipogenesis (DNL) may not have been affected, or even enhanced. At this time, the liver will convert the excess glucose into fatty acids, which are then esterified to form triglycerides (TG), leading to an excessive accumulation of triglycerides in the liver and causing hepatic steatosis (7, 8). In addition, insulin resistance also increases fat breakdown in adipose tissue, releasing a large amount of free fatty acids (FFAs) into the liver, further exacerbating the synthesis and accumulation of triglycerides within the liver (13). These excessive free fatty acids not only directly cause hepatic steatosis but also activate inflammatory pathways and oxidative stress within the liver,which in turn exacerbate hepatocyte damage and insulin resistance (13–16).

Insulin resistance not only leads to simple hepatic steatosis but is also a key driving factor for the progression of MASLD to MASH and liver fibrosis. Under the state of insulin resistance, the accumulated fat in the liver generates lipotoxicity, which activates Kupffer cells and other immune cells in the liver, causing them to release pro-inflammatory cytokines and chemokines such as TNF-α and IL-6, thereby triggering liver inflammation (17–19). These inflammatory factors, in turn, aggravate liver cell damage and insulin resistance. Excessive fatty acid oxidation and mitochondrial dysfunction also lead to an increase in reactive oxygen species (ROS) production, causing oxidative stress, which not only directly damages liver cells but also activates inflammatory and fibrotic signaling pathways. It is the interaction among lipotoxicity, inflammation and oxidative stress that jointly leads to programmed cell death (PCD) in hepatocytes. The damage-associated molecular patterns (DAMPs) released by hepatocyte death further activate Kupffer cells and hepatic stellate cells (HSCs), intensifying the inflammatory and fibrotic processes, and ultimately leading to liver fibrosis (20–22).

Therefore, accurately assessing the state of insulin resistance holds significant practical value for the early identification, risk stratification, and formulation of targeted treatment intervention plans for MASLD.

**Figure 1:**
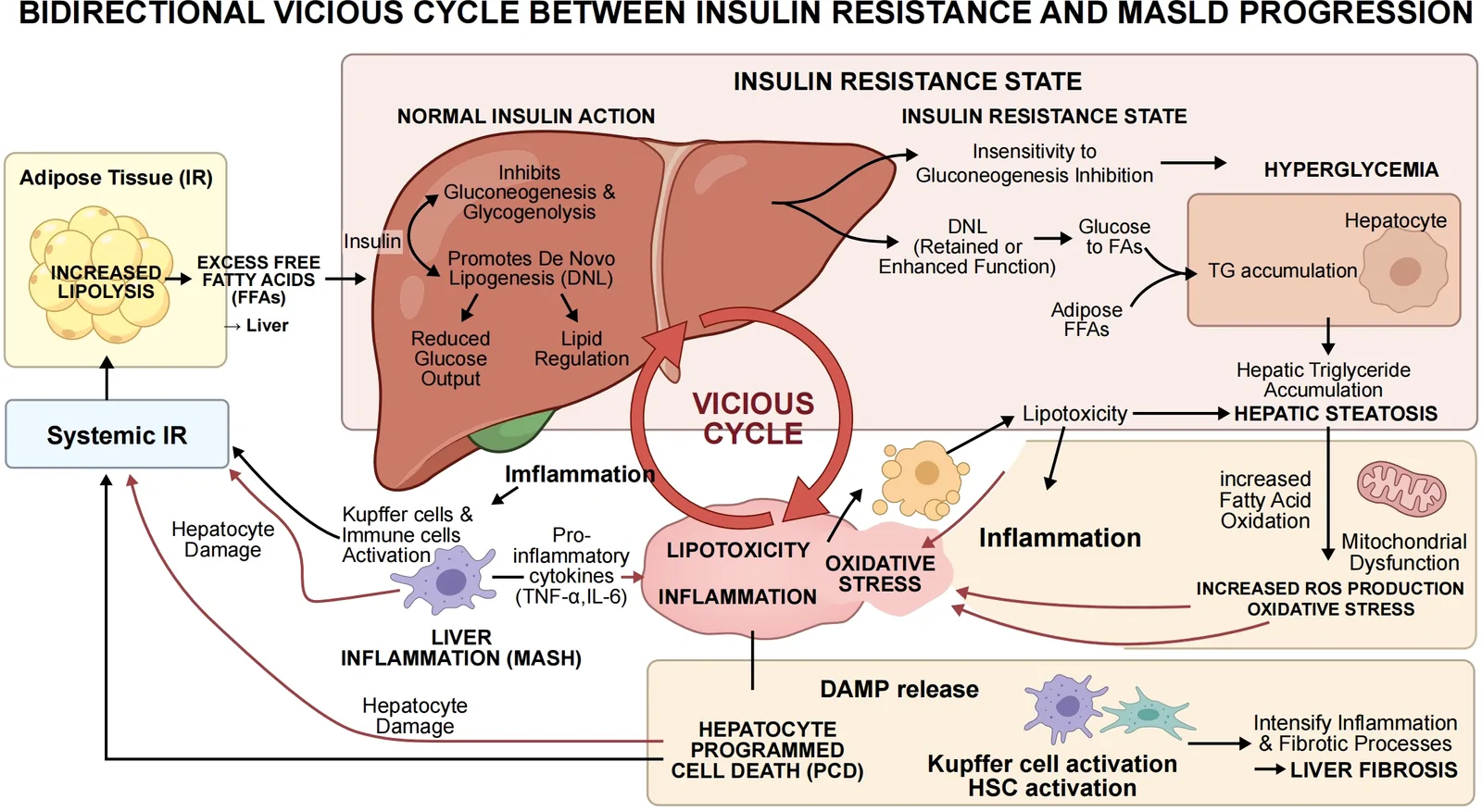
Bidirectional vicious cycle between insulin resistance (IR) and the progression of metabolic dysfunction-associated steatotic liver disease (MASLD).

The mechanistic pathways linking insulin resistance to the progression of metabolic-associated fatty liver disease are shown in **Figure 1**.

## 4 Application of insulin resistance-related indicators in the clinical evaluation of MASLD

Although the hyperinsulinemic-euglycemic clamp (HEC) technique is the “gold standard” for assessing insulin resistance, it is difficult to be widely adopted in clinical practice. In light of this reality, researchers have developed many alternative markers - these markers are based on routine biochemical indicators, which are non-invasive, economical, and easy to obtain, and can indirectly evaluate the state of insulin resistance. At the same time, researchers are also exploring their practical application value in the clinical assessment of MASLD.

### 4.1 HOMA-IR

HOMA-IR is a classic surrogate marker of insulin resistance calculated from fasting glucose and fasting insulin levels, using the formula: HOMA-IR = FPG (mmol/L) × FINS (mIU/L)/22.5 or FPG (mg/dL) × FINS (μIU/mL)/405 (23). This index shows a significant correlation with the gold standard for assessing insulin resistance—the hyperinsulinemic-euglycemic clamp test (HEC), with a correlation coefficient of r = -0.820 after log transformation (P < 0.0001) (24). HOMA-IR has demonstrated robust diagnostic utility for MASLD. In a 2010 study by Salgado et al. involving a non-diabetic population in Brazil, when HOMA-IR was set at a cutoff of 2.0, the area under the ROC curve (AUC) for MASLD diagnosis reached 0.840 (95% CI: 0.781–0.899), with sensitivity and specificity of 85% and 83%, respectively (25). Moreover, HOMA-IR is closely associated with disease progression in MASLD, particularly liver fibrosis. A cross-sectional study of Egyptian patients found that HOMA-IR is an independent predictor of progressive liver fibrosis (F≥2) in MAFLD patients, and this association is not affected by the presence or absence of diabetes or obesity (23). Multiple studies further support the view that the higher the HOMA-IR level, the greater the risk or severity of liver fibrosis in patients tends to be (26, 27). Fouad et al.’s study in 364 Egyptian MASLD patients further confirmed that in non-diabetic individuals, each unit increase in HOMA-IR was associated with a 14% higher risk of fibrosis, and the predictive performance was significantly better than in diabetic patients (adjusted OR = 1.03), suggesting that the diabetic state may impair HOMA-IR’s predictive power (23).

Beyond its association with liver fibrosis, HOMA-IR also demonstrates prognostic utility for cardiovascular outcomes in patients with MASLD (28). A 2022 meta-analysis of 19 prospective cohort studies (N = 161,032) reported that elevated HOMA-IR is significantly associated with a higher risk of non-fatal major adverse cardiovascular events (MACE), whereas no statistically significant association was observed with fatal MACE. The authors attributed the null finding for fatal MACE to limited statistical power—likely stemming from relatively short median follow-up duration (≈5.2 years) and low incidence of fatal events across included cohorts (29). In 2025, findings from a large prospective community-based cohort study in China (n = 8,035) were reported, demonstrating that HOMA-IR accounts for 31.07% of the total association between MASLD and incident atherosclerotic cardiovascular disease (ASCVD), a proportion significantly greater than that attributable to high-sensitivity C-reactive protein (hs-CRP; 14.70%). These results suggest that insulin resistance—quantified by HOMA-IR—may serve as a central pathophysiological mediator linking hepatic metabolic dysfunction to elevated ASCVD risk (30).

In terms of therapeutic monitoring, A single-center prospective observational study conducted in Italy in 2020 enrolled 65 obese patients with MASLD (45 completed the study). All participants received a 90-day stepped dietary intervention consisting of a very low-calorie ketogenic diet (VLCKD) followed by a low-calorie low-carbohydrate diet (LCD). Using a baseline HOMA-IR cutoff of 4.5, patients with baseline HOMA-IR ≥ 4.5 showed a significantly greater reduction in the hepatic steatosis index (HSI) after intervention than those with HOMA-IR < 4.5. Moreover, the magnitude of HOMA-IR reduction was significantly positively correlated with the improvement in hepatic steatosis (r=0.386, P = 0.002) (31).

In addition, some medications, such as GLP-1 receptor agonists and PPAR agonists, have also been proven to have a positive impact on MASLD by improving insulin resistance and reducing HOMA-IR (32–34).

It is noteworthy that the diagnostic cutoff values for HOMA-IR exhibit significant heterogeneity across populations and require stratification by geographic region, diabetes status, and obesity status. For example, by geographic region: male subjects in the Middle East (Iran) have a cutoff of 1.79 and females 1.95 (35); Italian populations show a value of 2.88 (36); and Brazilian populations 2.0 (25). By diabetes status: approximately 1.9 for non-diabetic individuals, 2.8–2.9 for high-risk mixed groups, and up to 4.5 for diabetic individuals (35), and the predictive performance of HOMA-IR is reduced in the diabetic population (23). However, systematic research on the heterogeneity of optimal cutoff values across different ethnicities and diabetes statuses remains limited. Most existing studies are cross-sectional in design, lacking large-scale prospective cohort validation, thus precluding causal inference. Furthermore, inadequate control for confounding factors such as obesity and diabetes in some studies has also affected the stability of their results.

### 4.2 TyG and its derived indicators

The triglyceride-glucose index (TyG) has emerged as a promising new marker for assessing insulin resistance in recent years. It can be calculated using only fasting triglycerides (mg/dL) and fasting glucose (mg/dL), with the formula TyG = ln (fasting TG × fasting glucose/2), making it simple, practical, and highly suitable for primary healthcare settings and large-scale population screening (37). In diagnosing MASLD, the overall performance of the TyG index is comparable to that of HOMA-IR (38), while derived indices—particularly TyG-BMI, which incorporates body mass index—demonstrate superior accuracy (39). A study conducted in 2024 involving a population at high altitudes (n=1384) demonstrated that the AUC of TyG-BMI for diagnosing MASLD was as high as 0.883 (40). A large-scale physical examination cohort study involving over 70,000 people in 2025 further confirmed that the diagnostic efficacy of TyG-BMI (AUC = 0.92) was significantly superior to that of the basic TyG index (AUC = 0.83) (37). Additionally, two other derived indices—TyG-WC (incorporating waist circumference) and TyG-WHtR (incorporating waist-to-height ratio)—have also been shown to help identify hepatic steatosis and fibrosis in patients with metabolic syndrome (41, 42). Analysis of data from nearly 100,000 individuals in the UK Biobank revealed that MASLD patients in the highest quartile of TyG-WHtR had a 39% higher risk of cardiovascular events, a 37% higher risk of all-cause mortality, and a 57% higher risk of cardiovascular death (43). A 2024 Italian study (involving 772 participants, 82.8% of whom had MASLD) also confirmed the predictive value of the TyG index for cardiovascular disease (AUC = 0.630), with a hazard ratio (HR) of 2.44 after multivariable adjustment (36).

Notably, diagnostic cutoff values for the TyG series show significant heterogeneity across populations. For example, the odds ratio (OR) linking TyG-BMI to MASLD was 1.07 in Chinese populations, but reached as high as 5.04 in U.S. populations (44). However, this latter result has a wide confidence interval, lacks statistical significance, and stems from few included studies with high heterogeneity, thus not reflecting true association strength and requiring further validation. Gender differences are also evident: the AUC for TyG-BMI in diagnosing MASLD was 0.88 in women, significantly higher than 0.83 in men (45). Regarding obesity status, TyG-WC was associated with increased risk of severe infection only among non-obese MASLD patients (aHR = 1.003), showing no significant link in obese individuals (46). It should be noted that most validation studies to date have focused on East Asian populations, limiting the generalizability of these indices to other ethnic groups.

### 4.3 TG/HDL-C ratio(triglyceride to high-density lipoprotein cholesterol ratio

The TG/HDL-C ratio, defined as fasting triglycerides divided by high-density lipoprotein cholesterol, primarily reflects lipid metabolism disorders closely associated with insulin resistance—specifically, hepatic triglyceride accumulation and reduced HDL-C levels, both of which are hallmark features of insulin resistance and MASLD (47, 48). In terms of the diagnosis of MASLD, multiple studies have reached a basic consensus that the TG/HDL-C ratio has a relatively good recognition value. One study showed that its diagnostic efficacy (AUC = 0.721) was similar to that of the TyG index (36). A study involving nearly 6,000 individuals from southwestern China found that the area under the curve (AUC) for this ratio was 0.795, when using a cutoff value of 1.09, sensitivity and specificity were 67.9% and 75.5%, respectively. When combined with ALT, AST, and albumin in a predictive model, the diagnostic efficacy could be significantly improved to 0.890 (95% CI: 0.882 - 0.898) (49). However, regarding whether this ratio can predict liver fibrosis, the existing research conclusions are not consistent and even divergent. Some studies, after adjusting for confounding factors, found no significant association between the ratio and liver fibrosis in adults (50, 51), while others suggest it holds some predictive value in pediatric populations (51). These discrepancies may stem from various factors, including differences in fibrosis assessment methods (transient elastography vs. liver biopsy), sample size variations (ranging from 376 to 5,220 participants), and differences in diabetes or obesity prevalence across study cohorts. Further research is needed to clarify these findings. Additionally, several studies support the role of this ratio in assessing cardiovascular risk among MASLD patients. There is evidence suggesting that even after adjusting for confounding factors, TG/HDL-C remains associated with the risk of major adverse cardiovascular and cerebrovascular events in patients with coronary heart disease (48). A study in 2024 further clarified that in patients with NAFLD combined with coronary heart disease, the TG/HDL-C ratio was positively correlated with the severity of coronary artery stenosis. For every one-unit increase in the ratio, the Gensini score, which reflects the degree of stenosis, increased by an average of 7.75 points (β = 7.75, 95% CI: 5.35 - 10.15, P < 0.001) (52).

Overall, the key advantage of the TG/HDL-C ratio lies in its simplicity and ease of measurement. However, its limitation is relatively low specificity, as it mainly reflects lipid metabolism and fails to capture the broader metabolic abnormalities involved in insulin resistance. In terms of therapeutic monitoring, the ratio can indicate improvements in lipid metabolism following intervention, but its utility in tracking histological improvement in liver tissue remains unclear.

### 4.4 METS-IR

METS-IR is a relatively new surrogate marker for insulin resistance that integrates multiple metabolic parameters, including fasting glucose, triglycerides, high-density lipoprotein cholesterol (HDL-C), and body mass index (BMI), aiming to provide a more comprehensive reflection of insulin resistance status. The formula for METS-IR is: METS-IR = ln((2 × fasting glucose) + triglycerides) × BMI/ln(HDL-C) (53). Research has confirmed that this indicator has a significant correlation with the gold standard of insulin sensitivity (hyperinsulinemic-euglycemic clamp test) (ρ = -0.622, P < 0.001), laying a methodological foundation for its clinical application (54). In diagnosing MASLD, METS-IR has demonstrated excellent performance across diverse populations. In 2018, it was first confirmed in a Mexican population that the area under the ROC curve (AUC) for diagnosing IR was 0.84 (95% CI: 0.78 - 0.90), with a sensitivity of 85.12% and specificity of 75.2% when the cut-off value was > 51.13 (54). A large Korean cohort study in 2022 found that METS-IR had an AUC of 0.824 in predicting MASLD prevalence, comparable to HOMA-IR (0.831), but significantly outperformed HOMA-IR in predicting MASLD incidence (55). A 2024 study based on the U.S. NHANES database further confirmed that METS-IR achieved a diagnostic AUC of 0.831, higher than HOMA-IR’s 0.767, in this study, METS-IR ≥ 40 was identified as a high-risk marker for MAFLD, and each unit increase in METS-IR was associated with approximately a 16% higher risk of MAFLD in the general population. Notably, the prevalence of MAFLD among Hispanic individuals was significantly higher than in other racial groups (56). In the pediatric and adolescent population, a 2025 South Korean study also demonstrated that its diagnostic efficacy (AUC = 0.91) was significantly superior to that of the TyG index (AUC = 0.69) and TG/HDL-C (AUC = 0.71) (57).

Beyond diagnosis, METS-IR also demonstrates significant value in assessing disease progression and prognosis. Multiple studies consistently show that METS-IR is closely associated with liver fibrosis risk and plays a key mediating role in the relationship between body fat percentage and liver fibrosis (58). Particularly noteworthy is its strong predictive power for long-term outcomes in MASLD patients. A 2024 study revealed that, after adjusting for confounding factors, METS-IR was the only insulin resistance indicator significantly associated with both all-cause mortality and cardiovascular mortality (hazard ratio (HR) for all-cause mortality = 1.015, HR for cardiovascular mortality = 1.018), whereas the TyG index and HOMA-IR were associated with all-cause mortality but not significantly linked to cardiovascular mortality (53). In terms of cardiovascular outcomes, the 2023 Chinese elderly cohort study demonstrated that for every quartile increase in METS-IR, the risk of cardiovascular disease in MASLD patients increased by 38%, and the risk of new-onset stroke increased by 51% (59).

In summary, current research widely recognizes METS-IR as a robust and highly effective metabolic assessment tool, valuable not only for diagnosing and risk stratifying MASLD but also for predicting liver fibrosis progression, cardiovascular events, and all-cause mortality. However, existing evidence primarily comes from observational studies, and there remains no prospective interventional trial confirming whether METS-IR can serve as a valid surrogate endpoint for treatment response. Moreover, the diagnostic cutoff values for this indicator exhibit significant heterogeneity across populations: for instance, the cutoff value for diagnosing MASLD in Italian inflammatory bowel disease patients was 36.52, whereas the threshold for insulin resistance in Mexican populations was 51.13 (23, 37, 60, 61); when stratified by body mass index (BMI), the association inflection point was 36 for individuals with a BMI below 25, compared to 46.73 in U.S. populations (23, 60, 62). Therefore, further in-depth exploration is needed regarding the optimization of METS-IR cutoffs in clinical practice, its generalizability across populations, and validation in interventional studies.

## 5 Challenges in the application of insulin resistance-related indicators

Insulin resistance-related indicators have substantial value in the clinical assessment of MASLD, however, several challenges remain before their widespread implementation in routine practice.

First, the lack of standardization remains a major barrier. Most currently available IR surrogate markers lack universally accepted calculation methods and diagnostic cut-off values, while population-specific characteristics—including race, age, sex, body mass index, and diabetes status—can substantially influence their normal ranges and threshold performance (63). In addition, no standardized population-stratified application framework currently exists, making precise clinical implementation difficult. Therefore, establishing standardized threshold libraries through multicenter studies across different ethnic and geographic populations is an important future priority.

Second, most IR-related indicators are surrogate markers derived from routine biochemical parameters rather than direct measurements of insulin sensitivity. As a result, they are susceptible to physiological variation and multiple confounding factors, including diet, physical activity, circadian rhythms, stress, and medication use, which may reduce their ability to accurately reflect true insulin resistance status (13, 64). More importantly, many IR markers primarily reflect systemic metabolic dysfunction rather than liver-specific insulin resistance, limiting their pathophysiological specificity for MASLD (23).

Third, important challenges remain regarding clinical application. Current IR markers may have limited ability to distinguish simple steatosis from MASH or accurately stage liver fibrosis severity. Furthermore, because MASLD is a dynamic disease process, whether longitudinal changes in IR markers can reliably reflect disease progression or treatment response remains unclear, highlighting the need for long-term prospective validation studies.

## 6 Conclusions

IR is closely related to MASLD. It aggravates the condition of MASLD through multiple mechanisms and increases the incidence of complications and mortality. Therefore, by assessing the IR status, not only can the severity and progression of MASLD be determined, but it also helps in the early diagnosis and prevention of the disease. TyG and its derived indices, TG/HDL-C, and METS-IR, as indicators for measuring IR, have the advantages of being easy to measure, simple and effective, and practical, and have broad application prospects in clinical practice. However, their application still needs to be continuously optimized and improved. Future research should focus on addressing the limitations of existing indicators, developing more accurate and non-invasive biomarkers, promoting individualized and precise diagnosis and treatment of MASLD, and ultimately improving the prognosis and quality of life of patients. To facilitate a clear and intuitive review of the advantages and disadvantages of the several IR markers mentioned above for the diagnosis and treatment of MASLD, we have created a table at the end of the article (**Table 1**).

**Table 1:** Comparison of advantages and disadvantages of common IR markers in the diagnosis and treatment of MASLD.

Indicator	Core advantages	Core limitations	Optimal application scenarios
HOMA-IR	Highly correlated with the hyperinsulinemic-euglycemic clamp test; shows significant value in diagnosing MASLD and predicting progressive liver fibrosis (F≥2); demonstrates stronger predictive performance in non-diabetic individuals; exhibits a mediating effect in the association between MASLD and ASCVD	The optimal cutoff values demonstrate significant population heterogeneity across racial groups, obesity statuses, and diabetes statuses; the predictive performance is diminished in obese populations.	Initial screening for fibrosis risk in non-diabetic MASLD patients; monitoring treatment response; bridging assessment of metabolic-cardiovascular risk
TyG and its derived indicators (TyG-BMI/TyG-WC/TyG-WHtR)	The calculation relies solely on conventional lipid and glucose parameters; TyG-BMI demonstrates superior diagnostic performance; TyG-WHtR and TyG-WC further enhance the assessment of obesity and prognosis.	Population heterogeneity is evident, with thresholds influenced by gender, race, and obesity status; the predictive value of some derived indicators decreases in obese subgroups; current validations have primarily focused on East Asian populations.	Primary care and large-scale screening; initial screening for MASLD in resource-limited settings; assessment of fatty liver risk in individuals with metabolic syndrome
TG/HDL-C	Rapid initial screening; risk assessment for patients with MASLD and coronary heart disease	Insufficient evidence for predicting adult liver fibrosis; primarily reflects lipid metabolism abnormalities, with limited coverage of systemic insulin resistance characteristics.	Rapid initial screening; risk assessment for patients with MASLD and coronary heart disease
METS-IR	Stable diagnostic performance across multiple metabolic parameters; the only IR indicator independently predicting both all-cause and cardiovascular mortality; outstanding diagnostic efficacy in children and adolescents; closely associated with fibrosis, stroke, and cardiovascular risk	Diagnostic thresholds show significant population differences; evidence is primarily derived from observational studies; generalizability across different racial groups and BMI categories still needs validation.	MASLD diagnosis and risk stratification; long-term prognosis assessment; screening in children/adolescents; prediction of fibrosis progression risk

## 7 Expert opinion

### 7.1 Impact on real-world outcomes

We believe that insulin resistance (IR) indices—including the Homeostatic Model Assessment of Insulin Resistance (HOMA-IR), the Triglyceride-to-HDL Cholesterol Ratio combined with Body Mass Index (TyG-BMI), and the Metabolic Score for Insulin Resistance (METS-IR)—hold significant potential in improving the real-world diagnosis and management of metabolic dysfunction-associated steatotic liver disease (MASLD). These non-invasive markers rely on routinely available clinical parameters such as fasting glucose, triglycerides, high-density lipoprotein cholesterol, and body mass index, making them particularly suitable for primary care settings and large-scale population screening, especially in resource-limited regions where advanced imaging or liver biopsy is not widely accessible.

By identifying individuals at high risk of progressing to metabolic dysfunction-associated steatohepatitis (MASH) and advanced liver fibrosis, these indicators may facilitate earlier initiation of lifestyle interventions and pharmacological treatments. Moreover, growing evidence suggests that IR-related markers also predict the risk of cardiovascular events, type 2 diabetes, and chronic kidney disease, allowing clinicians to simultaneously assess both hepatic and systemic metabolic risks.

From a healthcare system perspective, widespread use of low-cost IR markers could reduce unnecessary specialist referrals, lower healthcare costs, and optimize resource allocation. We further suggest that integrating validated IR markers into electronic health record systems may support automated risk stratification, facilitating population-based management strategies and personalized clinical decision-making.

### 7.2 Key areas for improvement and solutions

We believe that several key issues remain to be addressed before IR-related metrics can be routinely incorporated into the clinical management pathway for MASLD.

First, current studies still primarily focus on liver-specific outcomes, with insufficient attention given to MASLD as a systemic metabolic disorder. We therefore recommend that future research further explore the associations between IR markers and extrahepatic outcomes—including cardiovascular disease, chronic kidney disease, and metabolic complications—to establish a more comprehensive risk assessment framework.

Second, standardized clinical implementation pathways have not yet been established. To improve clinical feasibility, we recommend adopting a two-step screening strategy. For high-risk individuals such as those with obesity, type 2 diabetes, or metabolic syndrome, initial screening could involve calculating METS-IR or TyG-BMI using routine biochemical and anthropometric parameters, followed by identification of at-risk individuals based on population-specific thresholds adjusted for race, age, sex, body mass index, and diabetic status. For those who screen positive, further noninvasive fibrosis assessment—such as the FIB-4 score and elastography—should be performed, along with individualized lifestyle or pharmacological interventions.

Moreover, we believe that continuous dynamic monitoring of IR markers may serve as a practical tool for evaluating the effectiveness of lifestyle interventions and metabolic therapies.

### 7.3 Potential of further research

We believe that well-designed prospective studies have the potential to generate high-quality evidence and address significant gaps in the current field.

Direct head-to-head comparisons of different IR-related metrics may help identify the most appropriate assessment tools across various clinical settings and disease stages. Prospective studies may further quantify the mediating role of insulin resistance in the causal pathway linking MASLD to extrahepatic complications, thereby reinforcing the value of IR metrics as tools for systemic metabolic risk assessment.

Additionally, establishing robust population-specific thresholds based on prospective cohorts could facilitate their inclusion in clinical guidelines and promote automated clinical application. These research findings may ultimately enable low-cost, non-invasive risk stratification in large populations, reduce unnecessary referrals and invasive testing, and improve long-term clinical outcomes.

### 7.4 Future direction of study

We believe that future research should focus on the following three methodological directions.

First, multicenter prospective cohort studies involving diverse racial populations are needed to standardize methods for calculating IR indices and defining outcome criteria.

Second, rigorous head-to-head comparisons within the same study cohort should be conducted to directly validate the predictive value of HOMA-IR, TyG-derived indices, TG/HDL-C ratio, and METS-IR for liver fibrosis progression, cardiovascular events, and mortality risk.

Third, advanced analytical strategies—including mediation analysis, machine learning approaches, and genetic adjustments (such as PNPLA3 gene variants)—should be incorporated to optimize threshold selection and develop dynamic, individualized prediction models.

We also emphasize that international collaboration will be crucial for harmonizing calculation methods and establishing universally applicable reference standards.

### 7.5 Evolution of the field in five years

We expect that over the next five years, MASLD risk assessment models will gradually shift from traditional approaches based on fixed cutoff values toward dynamic, individualized risk prediction models.

We anticipate that primary care settings will progressively adopt a two-step screening strategy: first, using validated IR metrics combined with population-specific thresholds for initial screening, followed by second-line non-invasive fibrosis assessment for screen-positive individuals. Machine learning models integrating IR markers, clinical parameters, and genetic information may also gradually replace conventional single-marker evaluation methods.

However, future progress in this field will depend not only on technological advances but also on achieving equitable and accessible implementation across healthcare systems with varying resource levels.

### 7.6 Five-year perspective

From a longer-term perspective, we envision that advances in multi-omics technologies, digital health tools, and longitudinal metabolic monitoring will further reshape the management of MASLD.

IR-related markers may no longer function as isolated surrogate markers but instead become integral components of broader metabolic health assessment frameworks integrating longitudinal metabolic trajectories, genetic risk information, and continuously updated clinical data. Such integration may facilitate personalized prediction of liver fibrosis progression and systemic complication risks, thereby advancing precision prevention strategies.

Nevertheless, challenges related to data interoperability, privacy protection, and accessibility in resource-limited settings remain major barriers to widespread implementation.

In the insulin-resistant state, the liver becomes insensitive to insulin-mediated suppression of gluconeogenesis, leading to hyperglycemia, while *de novo* lipogenesis (DNL) remains retained or even enhanced. Together with excessive free fatty acids (FFAs) released from insulin-resistant adipose tissue, this drives hepatic triglyceride accumulation and steatosis. Subsequent lipotoxicity, oxidative stress, and inflammation (MASH) trigger hepatocyte damage and programmed cell death (PCD), releasing damage-associated molecular patterns (DAMPs) that activate Kupffer cells and hepatic stellate cells (HSCs), thereby promoting liver fibrosis. Conversely, hepatic steatosis, inflammation, and fibrogenesis further exacerbate systemic insulin resistance, forming a self-reinforcing vicious cycle that drives MASLD progression.
